# Detecting possible glaucoma with only limited equipment: a crucial first step

**Published:** 2012

**Authors:** Heiko Philippin, Peter Shah, Matthew Burton

**Affiliations:** Head of postgraduate training and glaucoma specialist: Kilimanjaro Christian Medical Centre, Moshi, Tanzania. Email: philippin@gmx.de; Ophthalmologist and glaucoma specialist: NHS Foundation Trust Birmingham, UK, and Centre for Health and Social Care Improvement, University of Wolverhampton, School of Health and Wellbeing, UK. Email: Peter.shah@uhb.nhs.uk; Senior Lecturer, International Centre for Eye Health, London School of Hygiene and Tropical Medicine, London, UK.

**Figure:**
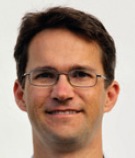
Heiko Philippin

**Figure:**
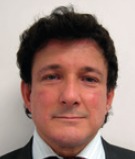
Peter Shah

**Figure:**
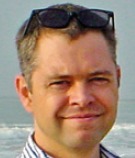
Matthew Burton

Glaucoma causes irreversible visual loss and must be detected as early as possible. It is therefore vital that all health care professionals are aware of glaucoma when they encounter patients who are most at risk. This includes people who are over 40 years of age and those who have first-degree relatives (siblings, parents or grown-up children) with glaucoma. People are also at higher risk if they wear spectacles for distance vision, if they have had an eye injury in the past, and if they complain about a gradual loss of vision.

There is no single test which can detect glaucoma; the key is to perform a basic eye assessment and to combine all findings to identify people who would benefit from a more comprehensive examination.

In some clinics, particularly at primary level, there is often only limited equipment available. However, by conducting the basic eye assessment described in this article, it will be possible to pick up important clues that suggest a patient is at high risk of glaucoma.

## 1 Take a history

Early glaucoma usually has no symptoms and the patient will be unaware of the problem. In advanced glaucoma, the person may describe a slow onset of visual loss and/or increasing difficulty avoiding obstacles, despite apparently still seeing well. This is because central vision is frequently preserved.

Ask every patient you see whether they have experienced such gradual loss of vision. Remember that many patients with loss of peripheral vision will not notice it until their glaucoma has become very advanced, and therefore difficult to treat. The visual field loss in each eye may also not be the same, allowing the person's brain to compose a complete image of the world by combining the two images from each eye (page 66). Some form of visual field testing for each eye separately is therefore advisable, such as confrontational field testing (see opposite and on page 69). You could also ask patients to close one eye at a time and tell you if they notice any differences in what they can see.

Primary open-angle glaucoma has a significant inheritable component and tends to be more common and more severe in people of African origin. A family history of the disease should raise suspicion of glaucoma, and everyone with a parent, brother, sister, or grown-up child with glaucoma should be referred for a comprehensive examination.

In Asia, angle-closure glaucoma is more common. Acute angle-closure is not only very painful but also can be associated with loss of vision and vomiting. Sometimes patients will have had episodes in the past which resolved, and so it is important to ask if they have ever experienced eye pain and headache which came on suddenly, with loss of vision or haloes around lights. Anyone with symptoms which suggest angle-closure glaucoma must be referred urgently for assessment to avoid permanent loss of sight.

**Figure 1:**
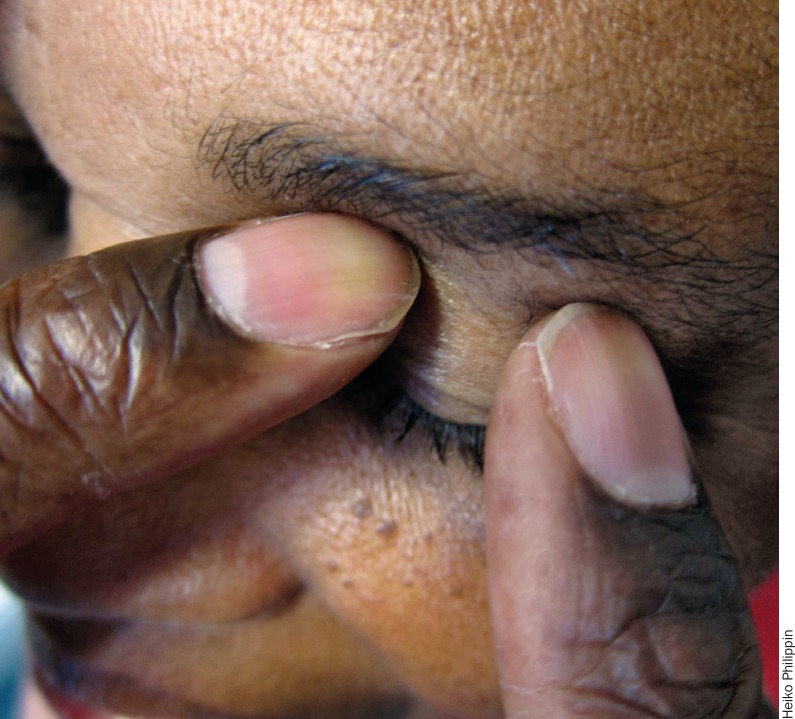
Palpating the eye carefully can help to identify very high intraocular pressure, a possible sign of glaucoma.

## 2 Observe the patient and talk to relatives

A person with advanced glaucoma may look and move differently. They may have obvious difficulty navigating and avoiding obstacles, especially in unfamiliar places.

People with extensive loss of visual field move slowly and carefully, often holding onto solid objects, such as the back of a chair, until they are certain that their next step is safe. People with advanced glaucoma with only a central island of preserved vision may have a staring gaze. Those with only limited residual peripheral vision may not look directly at your face when talking to you, but will look to the side, or tilt their head. They may find it difficult to see where the visual acuity chart is, for example, and may move their eyes or head around until the chart moves into their restricted field of view.

Relatives may report that the person finds it more and more difficult to move around, or takes a long time to find things, such as a piece of fruit on the table, even when it is quite obvious to others where the object is.

All these findings also can be caused by other diseases; however, everyone with such functional visual loss must be referred for a comprehensive eye examination.

## 3 Test visual acuity

This should always be tested with spectacles on if applicable.

A 1 m Snellen chart, or newspaper headlines, can be used at a distance of 6 m. Another option is to ask the patient to count fingers at different distances. The examiner can use his or her own visual acuity as a reference (assuming it is normal). Visual acuity is always tested for each eye independently.

In glaucoma, central vision, which is what is used for testing visual acuity, can be preserved until the disease is advanced. It is, therefore, vital to realise that **normal visual acuity does not exclude glaucoma.**

Also, loss of visual acuity can be due to many eye conditions, including refractive error and cataract. As a general rule, everyone with loss of visual acuity in one or both eyes must be referred so that the cause can be determined.

## 4 Confrontational visual field test

Advanced glaucoma is characterised by visual field defects (although not all visual field defects are due to glaucoma). Confrontational visual field testing (see page 69) can pick up marked visual field defects, particularly in patients whose visual acuity is still good. In confrontational visual field testing the examiner compares the patient's visual field with her or his own.

## 5 Assess digital intraocular pressure

Intraocular pressure (IOP) can be estimated by palpating the eye (Figure 1). Ask the patient to close her eyes and look down. Put the tips of both index fingers onto the closed upper eyelid. Keeping both fingertips in contact with the eyelid, apply gentle pressure through the closed upper eyelid, first gently pressing on the eye with the right index finger and then with the left, then with the right again (see picture opposite). A normal eye should feel a bit like a tomato that is just ripe: not solid, nor very soft. It is important to compare the two eyes with each other.

Although experience is needed to assess the intraocular pressure in this way and the method is not very accurate, a very high IOP may be detected as the eye feels abnormally hard and solid.^1^ This is a useful test if you suspect glaucoma from the history and from visual acuity testing. Every person in whom a high IOP is suspected should be referred for further examination.

**Figure:**
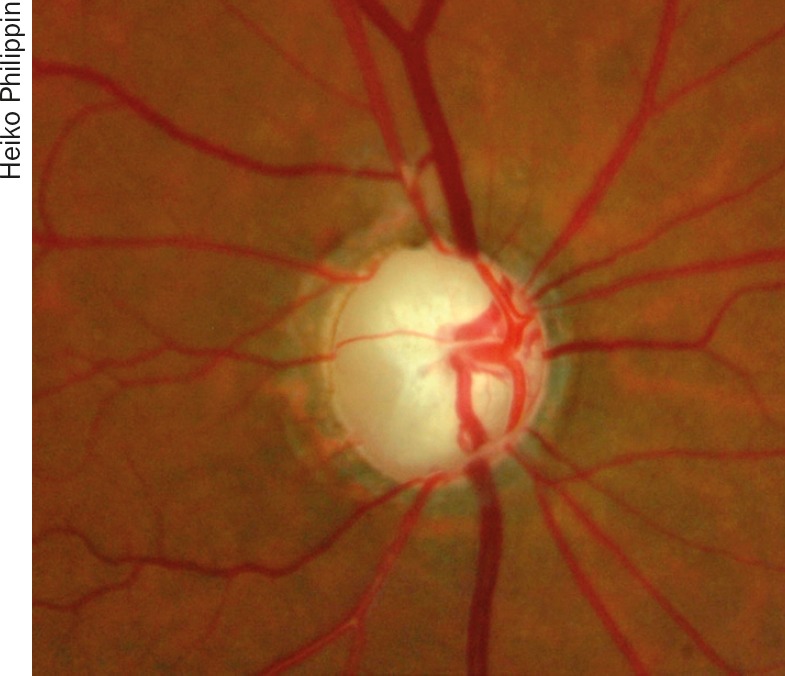
End-stage glaucoma

## 6 Use a torch

A simple hand-held torch can provide useful information about the appearance of the pupil, pupillary reactions to light, and the anatomy of the front of the eye.

### Pupil appearance

Use the torch to examine the front of the eye and look closely at the shape of the pupil and its response to light. An irregular, poorly responding pupil may suggest the presence of posterior synechiae from uveitis or an old injury, which can both lead to secondary glaucoma. This is sufficient reason to refer the patient for a more thorough eye examination.

### Exclude cataract

If the patient has loss of visual acuity in one or both eyes, and the pupil remains black, then cataract is probably not the cause of the vision loss: there must be another reason, such as glaucoma.

### Relative afferent pupillary defect

Although glaucoma usually affects both eyes, it is sometimes asymmetric. This can be detected with the relative afferent pupillary defect (RAPD) test – see article on page 58. As other diseases of the retina or optic nerve can also cause a RAPD (e.g. optic atrophy), all patients with RAPD should always be referred for further assessment. Remember: a RAPD is not only caused by a cataract.

## 7 Use a direct ophthalmoscope

Examination of the optic nerve head (ONH) is a **vital part** of the assessment for glaucoma, as this disease is characterised by changes in the ONH, which are subtle in the early stages of glaucoma. The ONH can be examined with a direct ophthalmoscope, preferably through a dilated pupil in a dimmed room. See the article on page 55 for guidance on how to examine the optic nerve head.

**NOTE**: Examination of the ONH with a direct ophthalmoscope can be challenging, for example if the pupil does not dilate well. If patients have a first degree relative with glaucoma, and particularly if they are over 40 or seem to have a visual field defect, you **must** refer them for further investigation even if you are unable to thoroughly assess the optic disc.

## 7 Educate and inform about glaucoma

After you have referred your patients, and treatment has been initiated, they may come back to you for possible lifelong follow-up. It is very important to talk to them repeatedly about the irreversible course of the disease, the need for lifelong control of their intraocular pressure, and the importance of regular follow-up visits. Many people are afraid of eye surgery, particularly when the ophthalmologist cannot promise them improved vision. Talk to patients about the benefits of surgery and, if possible, introduce them to other patients who have had successful glaucoma surgery.

**‘Glaucoma is challenging for eye care providers since it is not possible to improve vision’**

Glaucoma is challenging for eye care providers since it is not possible to improve the patient's vision. A lot of effort has to be invested in keeping the visual status stable in order to avoid blindness. Good communication and listening skills are critical if the patient is to understand their condition and adhere to a long-term treatment and follow-up plan.

If possible, patients with vision loss should be referred to an optometry or refractive error service to ensure they are making the best use of their vision. Some patients may require low vision support or community-based rehabilitation, so ensure that you are aware of these services and can make an appropriate referral.
